# The association between post-treatment surveillance testing and survival in stage II and III colon cancer patients: An observational comparative effectiveness study

**DOI:** 10.1186/s12885-019-5613-5

**Published:** 2019-05-03

**Authors:** Robert B. Hines, Md Jibanul Haque Jiban, Adrian V. Specogna, Priya Vishnubhotla, Eunkyung Lee, Shunpu Zhang

**Affiliations:** 1 Department of Population Health Sciences, University of Central Florida College of Medicine, 6900 Lake Nona Blvd, Orlando, FL 328270 USA; 2 University of Central Florida College of Health Professions and Sciences, Orlando, FL USA; 3 Orlando Veterans’ Affairs Medical Center, Orlando, FL USA

**Keywords:** Colon cancer, Surveillance, Survivorship, Quality of care, Comparative effectiveness research

## Abstract

**Background:**

The best strategy for surveillance testing in stage II and III colon cancer patients following curative treatment is unknown. Previous randomized controlled trials have suffered from design limitations and yielded conflicting evidence. This observational comparative effectiveness research study was conducted to provide new evidence on the relationship between post-treatment surveillance testing and survival by overcoming the limitations of previous clinical trials.

**Methods:**

This was a retrospective cohort study of the Surveillance, Epidemiology, and End Results database combined with Medicare claims (SEER-Medicare). Stage II and III colon cancer patients diagnosed from 2002 to 2009 and between 66 to 84 years of age were eligible. Adherence to surveillance testing guidelines—including carcinoembryonic antigen, computed tomography, and colonoscopy—was assessed for each year of follow-up and overall for up to three years post-treatment. Patients were categorized as More Adherent and Less Adherent according to testing guidelines. Patients who received no surveillance testing were excluded. The primary outcome was 5-year cancer-specific survival; 5-year overall survival was the secondary outcome. Inverse probability of treatment weighting (IPTW) using generalized boosted models was employed to balance covariates between the two surveillance groups. IPTW-adjusted survival curves comparing the two groups were performed by the Kaplan-Meier method. Weighted Cox regression was used to obtain hazard ratios (HRs) with 95% confidence intervals (CIs) for the relative risk of death for the Less Adherent group versus the More Adherent group.

**Results:**

There were 17,860 stage II and III colon cancer cases available for analysis. Compared to More Adherent patients, Less Adherent patients experienced slightly better 5-year cancer-specific survival (HR = 0.83, 95% CI 0.76–0.90) and worse 5-year noncancer-specific survival (HR = 1.61, 95% CI 1.43–1.82) for years 2 to 5 of follow-up. There was no difference between the groups in overall survival (HR = 1.04, 95% CI 0.98–1.10).

**Conclusions:**

More surveillance testing did not improve 5-year cancer-specific survival compared to less testing and there was no difference between the groups in overall survival. The results of this study support a risk-stratified, shared decision-making surveillance strategy to optimize clinical and patient-centered outcomes for colon cancer patients in the survivorship phase of care.

**Electronic supplementary material:**

The online version of this article (10.1186/s12885-019-5613-5) contains supplementary material, which is available to authorized users.

## Background

Recent studies indicate that approximately 20% of stage II and III colon cancer patients will have a recurrence of their cancer within 5 years following curative treatment (surgery ± adjuvant chemotherapy) [1–4]. In the US, guideline issuing groups such as the National Comprehensive Cancer Network (NCCN) have supported surveillance testing consisting of physical exams, carcinoembryonic antigen (CEA) blood tests, colonoscopy, and computed tomography (CT) scans of the chest and abdomen in the years following primary treatment [5–7]. The main purpose of colon cancer surveillance testing is to detect tumor recurrence or new primary colon cancer at an earlier point than symptom-based detection, resulting in a higher likelihood of curative treatment for the recurrence and better cancer-specific survival [8, 9]. However, although earlier randomized controlled trials (RCTs) and meta-analyses of these trials demonstrated that surveillance for local/regional colon cancer improved overall survival [10–13], the evidence that more intensive surveillance improves cancer-specific survival has not been demonstrated [9, 12–14]. This scenario has been complicated by the fact that the clinical trial data pertaining to this issue:1) has spanned a number of decades in which treatments for colon cancer have dramatically improved and surveillance strategies have evolved [15, 16], 2) includes heterogeneous studies with varying surveillance protocols that make comparisons between trials and inclusion of trials in meta-analyses problematic [15–19], and 3) has been derived from small controlled trials with limited follow-up, and thus, lacks the statistical power to detect meaningful survival differences [18, 19]. The resulting contradictory evidence has led some clinicians/researchers to question whether surveillance guidelines represent the best follow-up strategy and even if surveillance testing should be done at all [15, 20–22].

Observational comparative effectiveness research can provide high quality, nuanced evidence beyond that which may be feasible or ethical in RCT designs evaluating survival outcomes [23, 24]. This evidence can then be used to facilitate shared decision making between physicians and their patients [23–26]. The goal of this retrospective comparative effectiveness study was to assess the hypothesized survival benefit associated with more surveillance testing in stage II and III colon cancer patients following curative treatment. We utilized the National Cancer Institute’s (NCI) Surveillance, Epidemiology, and End Results database combined with Medicare claims (SEER-Medicare) to evaluate whether levels of adherence with NCCN Surveillance Guidelines (more vs. less) is associated with survival in older adult colon cancer patients. This study was designed to address the statistical power, heterogeneous surveillance strategies, and generalizability limitations of previous RCT designs by evaluating survival differences according to surveillance testing received in real world clinical settings. To achieve this objective, we sought to leverage the strengths of the large SEER-Medicare database with extensive follow-up time to evaluate 5-year cancer-specific survival and, secondarily, 5-year overall survival in colon cancer survivors according to levels of surveillance with NCCN guidelines.

## Methods

**Fig. 1:**
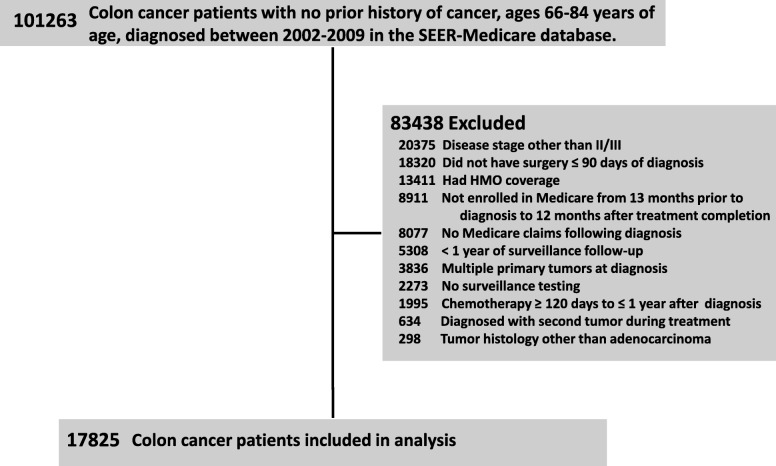
Flow diagram of the study population. SEER: Surveillance, Epidemiology, and End Results

Study methodology has been described although some differences will be presented herein [17]. The study population consisted of colon cancer patients, 66–84 years of age, who were diagnosed between 2002 to 2009 and included in the NCI’s SEER-Medicare database. These years were included to ensure that all patients had the potential for at least 5 years of follow-up after completion of treatment. Vital status and cause of death information was available up to the termination date of this study, December 31, 2015. Patients who received no surveillance tests (Nonadherent) for up to 3 years post-treatment were excluded. The flow diagram depicting inclusion/exclusion criteria is shown in Fig. 1.

### Ascertainment of study data

Diagnostic and procedure codes from the International Classification of Diseases, Ninth and Tenth Edition (ICD-9/10); International Classification of Diseases for Oncology, 3rd Edition (ICD-O-3); Current Procedural Terminology (CPT); and Healthcare Common Procedure Coding System (HCPCS) were utilized to obtain relevant treatment, surveillance, comorbidity [27, 28], and cause of death information (Additional file 1, Table S1).

### Final treatment date

For patients who received surgery as their only primary treatment, their final treatment date was their surgery date. For patients who received adjuvant chemotherapy, their final treatment date was the last date of sequential chemotherapy treatment (claims ≤90 days apart were considered as sequential chemotherapy) or 6 months from the first chemotherapy date for patients who received chemotherapy > 6 months.

### Surveillance tests

In determining adherence with NCCN Surveillance Guidelines, we considered receipt of surveillance tests that were established for their ability to detect new colon cancers and intraluminal, locoregional, and distant metastatic recurrences. Hence, we did not include physical exams in determining adherence with guidelines [19]. In each year of follow-up after the final treatment date, we obtained the number of CEA blood tests, CT (and positron emission tomography [PET]) scans, and colonoscopies. To avoid duplicate counting of surveillance tests, claims had to be separated by ≥30 days. We assessed adherence with surveillance tests up to 3 years post-treatment as this is the time period in which most recurrences occur [29]. In a given year, we defined adherence with a surveillance test as receiving the minimum number of recommended tests (Table 1) [5–7].

**Table 1:** NCCN Colon Cancer Surveillance Guidelines [30]

Stages II/III	CEA testing is recommended at baseline and at least every 6 months (minimum 2 per year) for 3 years. Colonoscopy is recommended approximately 1 year after resection. CT scans are recommended annually for 3 years.

Abbreviations: NCCN, National Comprehensive Cancer Network; CEA, carcinoembryonic antigen; CT, computed tomography

### Determining yearly and overall surveillance classification

For the assessment of yearly and overall surveillance, we modified our original strategy in order to capture the totality of the surveillance experience [17]. For CEA, which requires a minimum of two tests per year to meet minimum guidelines, we classified each year of follow-up according to the number of CEA tests as ≥2, 1, or 0. For tests in which the minimum was one test in a given year (Colonoscopy and CT), each patient was labeled as ≥1 or 0. For each year of surveillance assessment, we classified colon cancer cases as “More Adherent”, “Less Adherent”, or “Nonadherent”. Patients who were More Adherent either received all recommended testing, were missing one test, or missing more than one test but with additional testing (e.g., colonoscopy) that was absent for those who were Less Adherent. Less Adherent patients were missing at least one recommended tests without additional testing to compensate for the missing tests. Nonadherent patients received no surveillance tests during the assessment period. The classification scheme for yearly surveillance assessment is shown in Table 2.

**Table 2:** Classification scheme for yearly surveillance assessments

Year 1 categorization		Year 1 CEA^a^	Year 1 CT	Year 1 colonoscopy
More Adherent		**≥ 2**	**≥ 1**	**≥ 1**
		≥ 2	≥ 1	0
		≥ 2	0	≥ 1
		1	≥ 1	0
		1	0	≥ 1
Less Adherent		≥ 2	0	0
		1	0	0
		0	≥ 1	0
		0	0	≥ 1
Nonadherent		0	0	0
Year 2 categorization	Year 1 colonoscopy	Year 2 CEA	Year 2 CT	Year 2 colonoscopy
More Adherent		**≥ 2**	**≥ 1**	
		≥ 2	0	≥ 1
	≥ 1	≥ 2	0	0
	≥ 1	1	0	0
		1	≥ 1	
		1	0	≥ 1
Less Adherent	0	≥ 2	0	0
	0	1	0	0
		0	≥ 1	
		0	0	≥ 1
Nonadherent		0	0	0
Year 3 categorization	Year 2 colonoscopy	Year 3 CEA	Year 3 CT	Year 3 colonoscopy
More Adherent		**≥ 2**	**≥ 1**	
		≥ 2	0	
		1	≥ 1	
		1	0	≥ 1
	≥ 1	1	0	0
Less Adherent	0	1	0	0
		0	≥ 1	
		0	0	≥ 1
Nonadherent		0	0	0

Abbreviations: CEA, carcinoembryonic antigen, CT, computed tomography

Bold font indicates testing that is adherent with NCCN guidelines

In determining overall surveillance, all years of complete follow-up up to 3 years post-treatment were assessed, and patients were classified as More Adherent or Less Adherent (Nonadherent patients were excluded). For patients with only 1 year of complete follow-up, their Year 1 classification was also their overall classification. For patients with two or 3 years of complete follow-up, their yearly classifications were combined to create an overall classification. Thus, for patients with 2–3 years of complete follow-up, their yearly surveillance assessments determined their overall surveillance classification. The overall classification scheme for patients with two or 3 years of complete follow-up is contained in Additional file 2 (Table S2).

### Statistical analysis

Demographic and clinical variables for the unweighted study population were compared across the two categories of overall surveillance (More vs. Less). Pearson’s chi-square was applied to detect differences in categorical variables. Median follow-up time and 25% cancer death time between the two groups were calculated by the Kaplan-Meier method with differences evaluated by the log-rank test. Statistical significance was defined as *P* value < 0.05.

In observational comparative effectiveness studies—where treatment is not randomized—appropriate methods to control for potential differences in confounding variables between the treatment groups is paramount [31]. Generalized boosted models represent a relatively new, machine learning approach, whereby propensity scores are obtained via multiple regression trees that model complex associations between pretreatment covariates across categories of treatment (i.e., surveillance) [32–35]. This procedure produces inverse probability of treatment weighting (IPTW) that balances covariates between groups. Propensity scores for each treatment group were obtained by balancing covariates according to age, race, sex, marital status, year of diagnosis, state buy-in coverage, census-tract poverty level, urban-rural designation, SEER region [36], disease substage, tumor grade, tumor location (proximal/distal), chemotherapy status, and the individual comorbid conditions that comprise the Charlson Comorbidity Index [27]. Balance between potential confounding factors was assessed by the absolute standardized mean difference for each group compared to the study population mean. Differences ≥0.20 were considered as evidence of imbalance [32]. For point estimates obtained in the regression models described below, the estimand is the average treatment effect.

Five-year survival curves for the study population were obtained by the Kaplan-Meier method which incorporated IPTW. The weighted Cox proportional hazards model was used to obtain adjusted hazard ratios (HRs) with 95% confidence intervals (CIs) for the relative hazard (i.e., risk) of five-year colon cancer-specific, cancer-specific (i.e., any cancer death), noncancer-specific, and overall mortality from the last date of treatment. The survival analysis revealed nearly identical hazard ratios for colon cancer-specific and cancer-specific survival, thus, only cancer-specific mortality is reported. We initially planned to obtain separate models according to treatment (surgery, surgery + chemotherapy). However, when we analyzed both models, the associations between surveillance status and all survival outcomes were highly similar. Thus, all patients were combined. The proportional hazards assumption was assessed for surveillance status graphically (log [−log] of survival function by log survival time) and by evaluating the interaction with time in the survival models. Although there was slight evidence of a declining hazard ratio (moving to the null) over time for cancer-specific survival, there was not sufficient evidence to indicate a violation of the proportional hazards assumption.

## Results

Demographic and clinical characteristics of the study population (*n* = 17,825) are shown in Table 3. The majority of colon cancer patients were designated as More Adherent vs. Less Adherent (11,840, 66.3% vs. 6020, 33.7%). More Adherent patients were more likely to be alive at 5 years post-treatment, but also more likely to have died of cancer. Other variables that were significantly associated with surveillance status were age, race, marital status, year of diagnosis, state buy-in coverage, census tract poverty level, disease stage, tumor grade, receipt of adjuvant chemotherapy, and comorbidity. Sex, geographic residency, SEER region, and tumor site were not associated with overall surveillance status.

**Table 3:** Demographic and clinical characteristics^a^ of stage II/III colon cancer patients (*n* = 17,860)

Characteristic	More Adherent	Less Adherent	*P* value
Study population	11,840 (66.3)	6020 (33.7)	
Median follow-up time			< 0.001
(years), 95% CI	9.0 (8.9–9.0)	9.6 (9.4–9.7)	
5-year vital status			< 0.001
Alive	8229 (69.5)	3908 (64.9)	
Deceased	3611 (30.5)	2112 (35.1)	
Cause of death			< 0.001
Colon cancer	2133 (59.1)	787 (37.3)	
Other Cancer	333 (9.2)	206 (9.7)	
Other	1145 (31.7)	1119 (53.0)	
25% cancer death time			< 0.001
(years), 95% CI	5.3 (5.0–5.8)	7.1 (6.3–7.8)	
Age at diagnosis			< 0.001
66–74 years	5814 (49.1)	2114 (35.1)	
75–79 years	3417 (28.9)	1774 (29.5)	
80–84 years	2609 (22.0)	2132 (35.4)	
Race			< 0.001
White	10,047 (84.9)	5066 (84.2)	
Black	945 (8.0)	563 (9.3)	
Asian	421 (3.6)	163 (2.7)	
Other	242 (2.0)	97 (1.6)	
Hispanic	158 (1.3)	100 (1.7)	
Native American	21 (0.2)	25 (0.4)	
Unknown	6 (0.0)	6 (0.1)	
Sex			0.304
Female	6645 (56.1)	3330 (55.3)	
Male	5195 (43.9)	2690 (44.7)	
Marital status			< 0.001
Married or partner	6876 (58.1)	2915 (48.4)	
Separated/divorced	764 (6.5)	437 (7.3)	
Single	855 (7.2)	533 (8.8)	
Widowed	3009 (25.4)	1897 (31.5)	
Unknown	336 (2.8)	238 (4.0)	
Year of diagnosis			0.011
2002–2003	3411 (28.8)	1820 (30.2)	
2004–2006	4442 (37.5)	2304 (38.3)	
2007–2009	3987 (33.7)	1896 (31.5)	
State buy-in coverage			< 0.001
No	9574 (80.9)	4306 (71.5)	
Yes	2266 (19.1)	1714 (28.5)	
Census tract poverty level			< 0.001
Low	3298 (27.8)	1495 (24.8)	
Lower-middle	3290 (27.8)	1629 (27.1)	
Upper-middle	3227 (27.3)	1726 (28.7)	
High	1988 (16.8)	1150 (19.1)	
Unknown	37 (0.3)	20 (0.3)	
Geographic residency			0.473
Urban	10,318 (87.2)	5263 (87.4)	
Less Urban	1221 (10.3)	627 (10.4)	
Rural	299 (2.5)	129 (2.2)	
Unknown	2 (0.0)	1 (0.0)	
SEER region			0.483
West	4226 (35.7)	2214 (36.8)	
South	3089 (26.1)	1556 (25.8)	
Northeast	2599 (22.0)	1299 (21.6)	
Midwest	1754 (14.8)	878 (14.6)	
Pacific	172 (1.4)	73 (1.2)	
Disease stage			< 0.001
Stage II	5912 (49.9)	4091 (68.0)	
Stage III	5928 (50.1)	1929 (32.0)	
Tumor grade			< 0.001
Low grade	8811 (74.4)	4811 (79.9)	
High grade	2784 (23.5)	1107 (18.4)	
Unknown	245 (2.1)	102 (1.7)	
Tumor site			0.153
Proximal colon	7627 (64.4)	3943 (65.5)	
Distal colon	4213 (35.6)	2077 (34.5)	
Adjuvant chemotherapy			< 0.001
No	5518 (46.6)	4847 (80.5)	
Yes	6322 (53.4)	1173 (19.5)	
Charlson Comorbidity Index			< 0.001
0	5953 (50.3)	2539 (42.2)	
1	3229 (27.3)	1617 (26.9)	
2–3	2143 (18.1)	1428 (23.7)	
4 +	515 (4.3)	436 (7.2)	

^a^Categorical variables are reported as frequency, (%). Measures of time are reported as median/25%, (95% confidence interval [CI])

The assessment of balance for the unweighted and weighted study samples is depicted in Additional file 3 (Table S3). Before weighting, when compared to the study population, there was evidence of unbalanced data for both groups in terms of receipt of adjuvant chemotherapy. The Less Adherent group was also unbalanced for the 80 to 84 years age category and disease stage. After weighting, all covariates were balanced between groups.

**Fig. 2:**
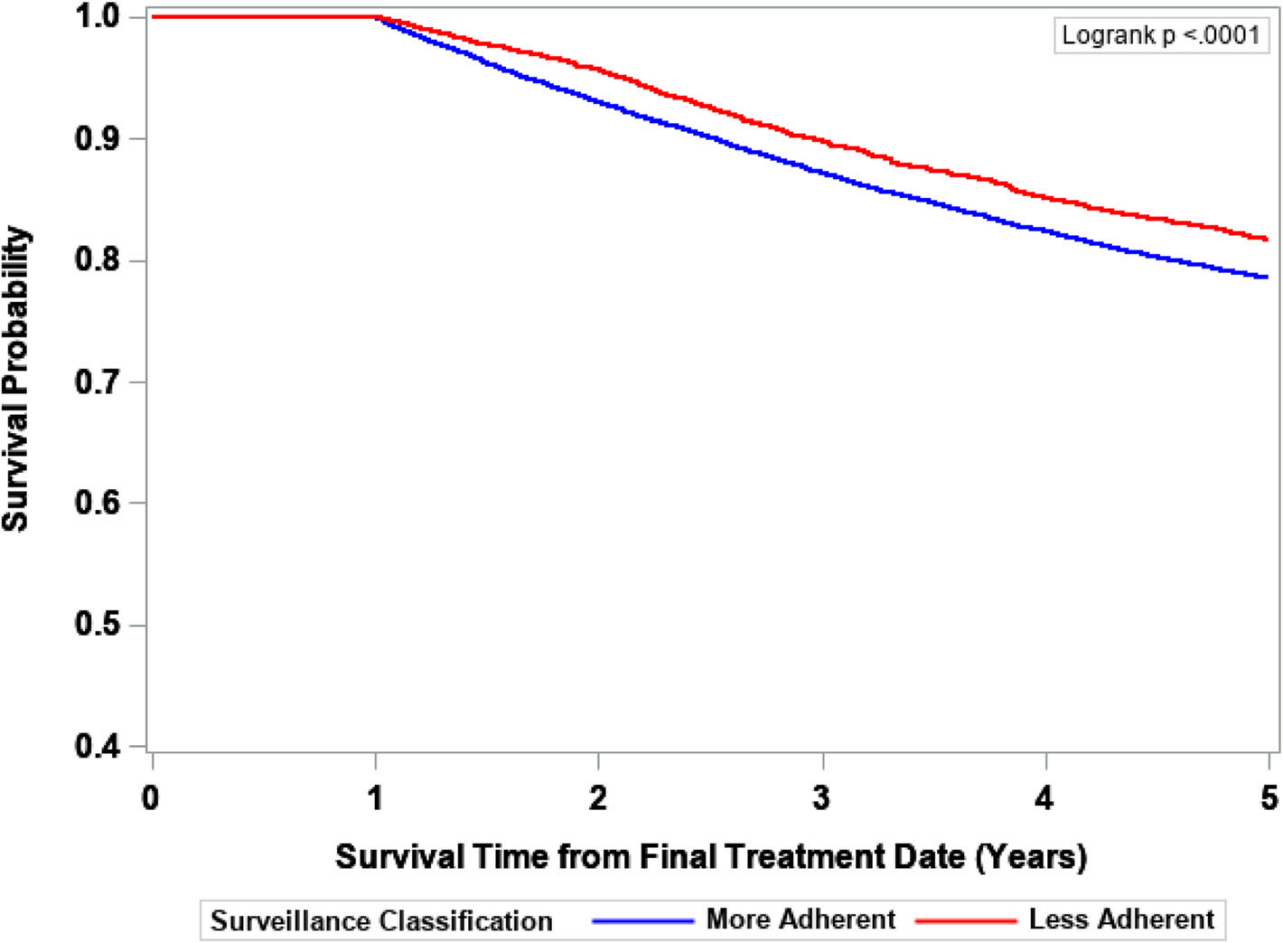
5-year cancer-specific survival probability by surveillance status. The Kaplan-Meier method was used to obtain IPTW-adjusted survival curves with statistical significance defined by the log-rank test

**Fig. 3:**
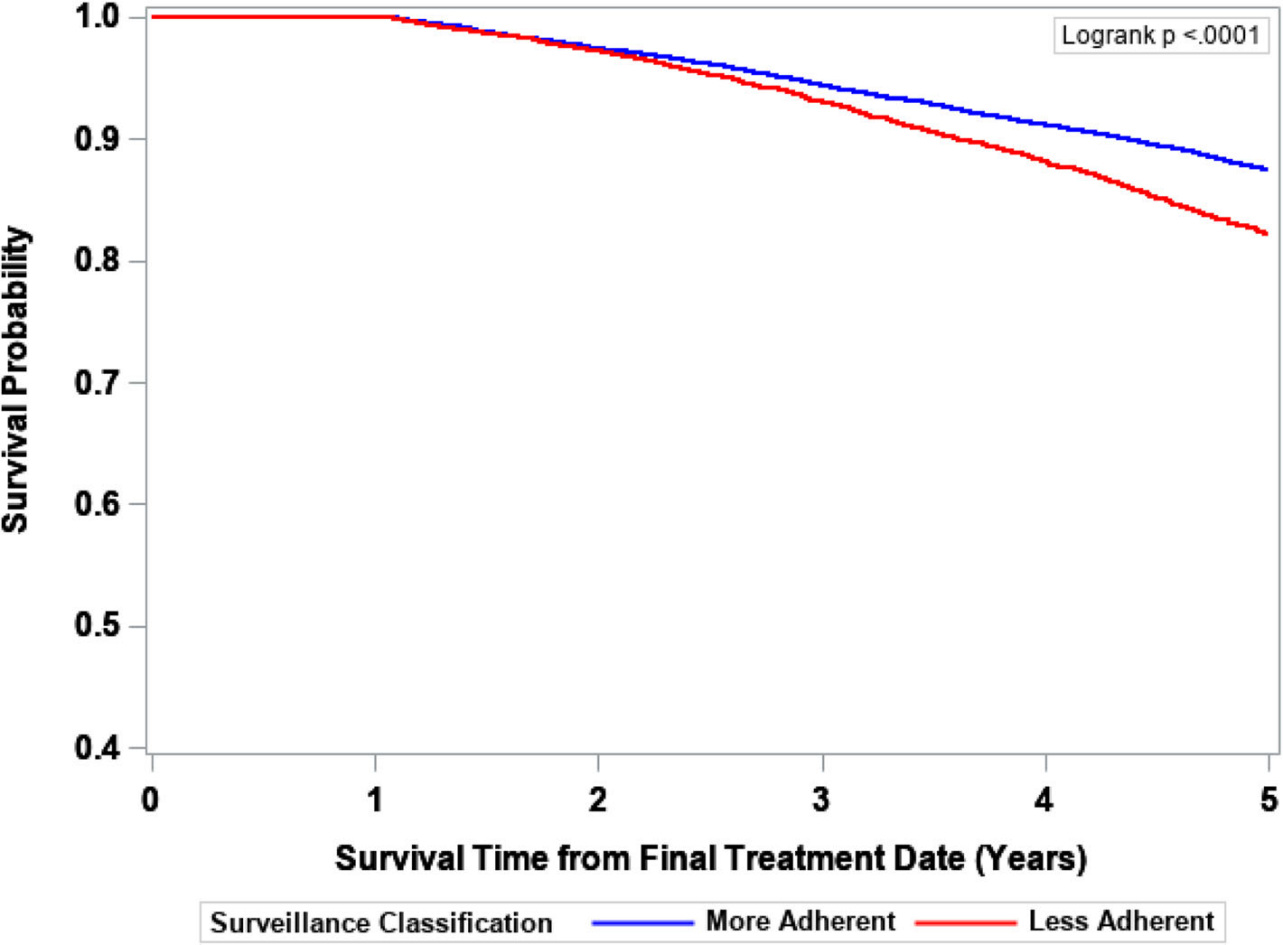
5-year noncancer-specific survival probability by surveillance status. The Kaplan-Meier method was used to obtain IPTW-adjusted survival curves with statistical significance defined by the log-rank test

**Fig. 4:**
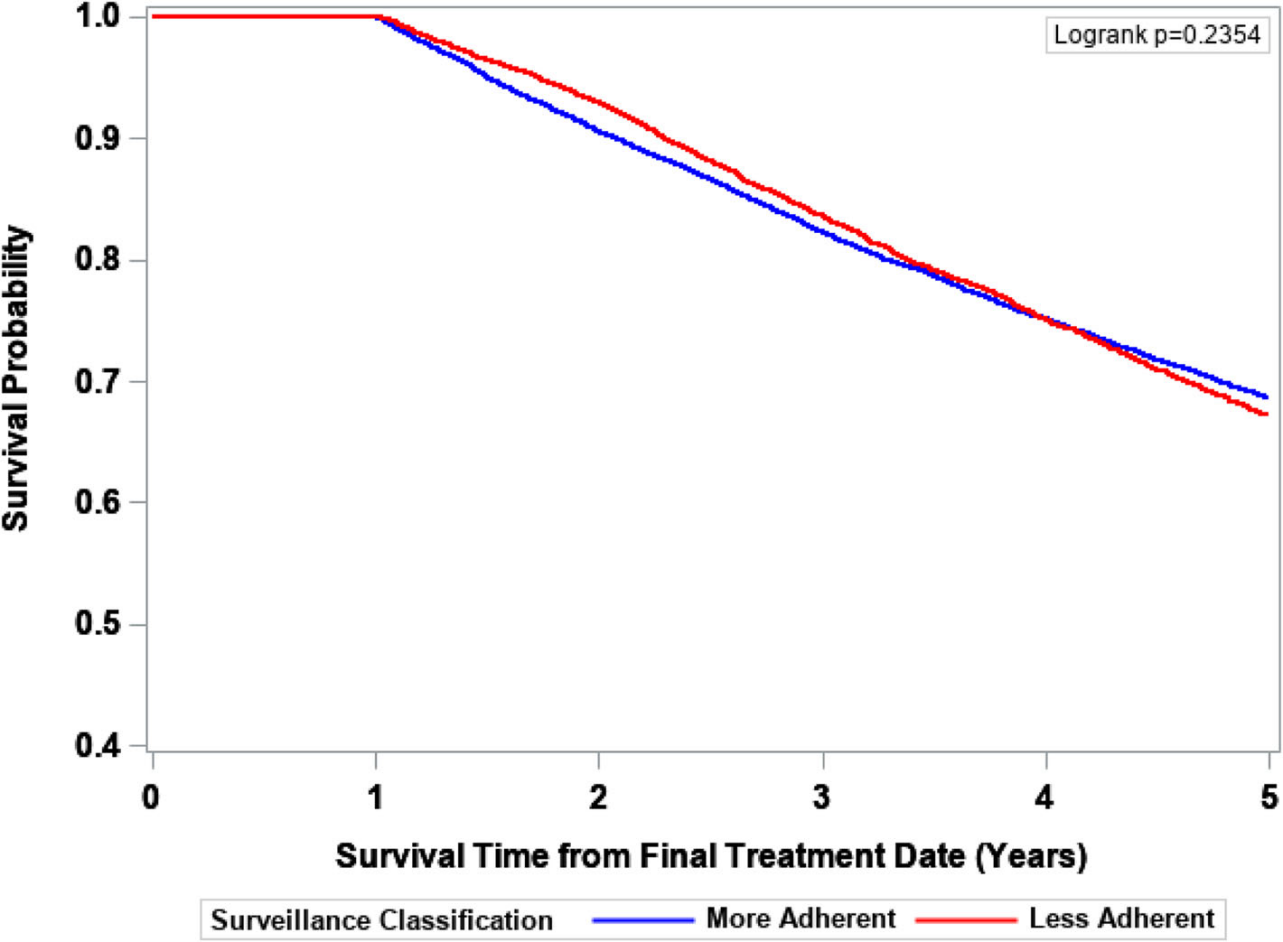
5-year overall survival probability by surveillance status. The Kaplan-Meier method was used to obtain IPTW-adjusted survival curves with statistical significance defined by the log-rank test

The IPTW-Kaplan-Meier survivor curves for 5-year cancer-specific, noncancer-specific, and overall survival are displayed in Figs. 2, 3, and 4, respectively. The More Adherent group experienced slightly poorer 5-year cancer-specific survival, better noncancer-specific survival for years 2–5, and no difference in 5-year overall survival. As reflected by the survival curves, the earliest death could not occur until 12 months after final treatment date due to inclusion/exclusion criteria (all patients had to have ≥1 year of surveillance follow-up).

Mortality data are provided in Table 4. Compared to colon cancer patients who were More Adherent, Less Adherent patients experienced a 17% decreased risk of 5-year cancer-specific death (HR = 0.83, 95% CI 0.76–0.90), and a 61% increased risk of 5-year noncancer-specific death (HR = 1.61 95% CI 1.43–1.82) that was limited to years 2 to 5. There was no difference between the groups in overall survival (HR = 1.04, 95% CI 0.98–1.10).

**Table 4:** IPTW-adjusted hazard ratios for the association between surveillance status and 5-year cancer-specific, noncancer-specific, and overall mortality

	5-year Cancer-specific death (events = 3459)^a^		5-year Noncancer-specific death (events = 1256)^a, b^		5-year Overall mortality (events = 5723)^a^	
Surveillance Status	HR (95% CI)	*P* Value	HR (95% CI)	*P* Value	HR (95% CI)	*P* Value
More Adherent	Ref		Ref		Ref	
Less Adherent	0.83 (0.76–0.90)	< 0.001	1.61 (1.43–1.82)	< 0.001	1.04 (0.98–1.10)	0.226

Abbreviations: IPTW, inverse probability of treatment weighting; HR, hazard ratio; CI, confidence interval

^a^The number of unweighted deaths

^b^For years 2–5

## Discussion

The controversy surrounding the use of surveillance testing in colon cancer survivors has been ongoing for several decades [37–39] as described in several meta-analyses which included studies published between 1995 to 2016 [12, 13, 21, 40–43]. Although guideline-issuing groups in the United States are consistent in their recommendation that stage II and III colon cancer patients receive surveillance testing following completion of treatment, we found that there is no benefit to more testing vs. less testing in terms of cancer-specific and overall survival in an older adult patient population. Future studies with more granular patient data (e.g., prognostic/predictive biomarkers) are needed to develop risk-stratified surveillance strategies with the goal of decreasing disease-related morbidity and mortality associated with recurrence.

Earlier RCTs on this topic were hampered by a number of limitations which resulted in conflicting evidence [15, 17]. Recently, higher quality studies have provided a more consistent conclusion regarding more vs. less surveillance testing. In 2014, Primrose et al. [44] published results of the Follow-Up After Colorectal Surgery (FACS) trial conducted in the UK. This study revealed that surveillance with CEA/CT increased the likelihood of curative resection by three times compared with minimal follow-up care, but no differences in survival were indicated. The results of two studies—one RCT and one observational study—were recently published in 2018 [2, 4]. Wille-Jorgensen et al. [2] reported the results of the multicenter COLOFOL trial which overcame many of the aforementioned limitations of earlier trials by boasting a large study population (*n* = 2555) with long-term follow-up. These investigators evaluated higher vs. lower frequency surveillance testing using CEA/CT. At the completion of the trial, there were no differences between the groups in either cancer-specific or overall survival. The only other observational study to evaluate more vs. less frequent surveillance testing was recently published by Snyder et al. [4]. This study differed from ours in a number of ways including: 1) surveillance was defined as high vs. low intensity CEA/CT testing at the facility level as opposed to individual-level assessment; 2) colon and rectal cancer patients were combined; 3) stage I patients (who only require colonoscopy to be adherent with guidelines) were included; 4) assessment of colonoscopy was not considered; and 5) differing inclusion/exclusion criteria were applied. This study, too, found no differences in overall survival according to facility-level surveillance.

Consistent with the results reported by the authors of the three aforementioned studies [2, 4, 44], in our study, patients who were More Adherent with guidelines did not experience improved cancer-specific survival compared to those who were Less Adherent. In fact, those who were Less Adherent with guidelines experienced slightly better 5-year cancer-specific survival. A reason for these seemingly contradictory results is possibly due to a study design limitation. Namely, as receipt of surveillance testing was not randomized, those who were deemed at greater risk for cancer recurrence/death received more surveillance testing. Similarly, patients in the Less Adherent group had a lower risk of cancer-specific death, but a higher risk of noncancer-specific death. These hypotheses are supported by the bivariable data in Table 3 and the mortality data in Table 4. Thus, due to a lower perceived risk of cancer-specific mortality and a higher risk of mortality from chronic comorbid conditions, patients in the Less Adherent groups underwent, appropriately, less surveillance testing and experienced slightly better cancer-specific survival. It is plausible that less encounters with the medical system in the Less Adherent group led to poorer control of comorbid conditions and an increased risk of noncancer-specific mortality. It is also possible that our assessment of comorbidity using the individual comorbid conditions in the Charlson Comorbidity Index [27] did not fully capture the comorbidity burden in our study population. Although our method of IPTW balanced all measured covariates between the two groups, there could have been additional factors related to comorbid disease burden that were not measured which may explain these results.

Our findings in an older adult colon cancer population indicate that there is considerable latitude regarding adherence with surveillance guidelines and the relationship with cancer-specific survival. Given the demonstration that treatment for cancer can result in financial (in addition to treatment-related) toxicity, lower cost/less intensive surveillance strategies might be attractive for many patients in the US and other first world countries, as well as in more resource-challenged environments [45–48]. Regardless of cost, shared decision making between patients and providers concerning the best surveillance strategy is appropriate to balance patient preferences, quality of life, suitability for curative treatment if recurrence is detected, risk of recurrence and likelihood of cure, and the presence of comorbid conditions which may present a much larger risk of short-term mortality [18, 49]. The purpose of comparative effectiveness research is to give patients and clinicians more information to inform these discussions [23–26]. Given the evidence from the current study and other recent investigations which have demonstrated the lack of cancer-specific and overall survival benefit with more surveillance testing, it may be appropriate to revisit guideline recommendations.

This study has a number of strengths. We leveraged the powerful SEER-Medicare database which contains demographic, comorbidity, tumor-related, treatment, follow-up, vital status, and cause of death information for cancer patients diagnosed in one of the 17 SEER regions in the US. The SEER-Medicare files contain an enormous amount of claims data with redundancies across file types. This helps to ensure the accuracy of information, which is a limitation of claims-based studies. Finally, we developed an individual-level classification scheme to assess surveillance testing for up to 3 years following treatment completion. We feel that our approach, which was based on obtaining an individual-level, holistic evaluation of surveillance reflecting what patients actually received, is a significant strength of this study.

These results should be interpreted with full consideration of the study’s limitations. The main limitation concerns judgments regarding comparative effectiveness using an observational study design. The gold standard for determining treatment efficacy is the RCT, but these designs are often not ethical (randomizing to receive no surveillance testing despite recommendations) or feasible due to the costs of enrolling a large number of patients with years of follow-up [23, 24]. Our study enabled the evaluation of a range of surveillance testing experiences reflecting what patients actually received. However, it should be acknowledged that although we achieved balance on the measured, potential confounders available in our dataset, unmeasured prognostic factors may have remained associated with surveillance status. Despite these limitations, the conclusions of this study remains valid. That is, more surveillance testing does not improve cancer-specific or overall survival compared to less testing.

A criticism of this study could relate to the surveillance classification scheme. Unlike a controlled trial, every variation of surveillance testing received in real world clinical settings had to be categorized and combined for up to 3 years of follow-up. Although one could possibly differ with the classification scheme used in this study, the study conclusions are the same. That is, more surveillance testing did not confer a cancer-specific or overall survival benefit compared to less testing. Another weakness of the study is that information on tumor recurrence cannot be observed via the SEER-Medicare database and the reason for testing is unavailable. Thus, it was not possible to differentiate true surveillance testing of asymptomatic patients from diagnostic testing in patients presenting with symptoms. Finally, our conclusions concerning the older adult/elderly colon cancer population may not be directly generalizable to younger patients.

## Conclusions

In a population of older adults with stage II and III colon cancer, more surveillance testing did not result in better cancer-specific or overall survival. These results support an individualized surveillance testing strategy that considers patients’ preferences, risk assessment of recurrence, and other individual-level clinical factors (e.g., comorbidity). In conjunction with results from recent studies [2, 4, 44], our findings may warrant a reconsideration of guidelines. At a minimum, the results of this study support shared decision making between older adult colon cancer patients and their healthcare providers. Efforts to ensure high quality cancer care which includes a patient-specific surveillance testing strategy are necessary to achieve the best clinical and patient-centered outcomes for stage II and III colon cancer patients in the survivorship phase of care.

## Additional files

Additional file 1: **Table S1.** ICD-O-3, ICD-9/10-CM, CPT, HCPCS, and Revenue Center Codes. (DOCX 13 kb)

Additional file 2: **Table S2.** Classification scheme for overall surveillance assessments. (DOCX 12 kb)

Additional file 3: **Table S3.** Unweighted and Weighted Means According to Adherence Status and the Unweighted Study Population. (DOCX 19 kb)

## Abbreviations

CEA: Carcinoembryonic antigen
CI: Confidence interval
COLOFOL: Assessment of Frequency of Surveillance after Curative Resection in Patients with Stage II and III Colorectal Cancer
CPT: Current Procedural Terminology
CT: Computed tomography
FACS: Follow-Up After Colorectal Surgery
HCPCS: Healthcare Common Procedure Coding System
HR: Hazard ratio
ICD-9: International Classification of Diseases Ninth Edition
ICD-O-3: International Classification of Diseases for Oncology 3rd Edition
IPTW: Inverse probability of treatment weighting
NCCN: National Comprehensive Cancer Network
NCI: National Cancer Institute
PET: Positron emission tomography
SEER: Surveillance, Epidemiology, and End Results
UK: United Kingdom
US: United States

## References

[1] Rosati G, Ambrosini G, Barni S, Andreoni B, Corradini G, Luchena G, Daniele B, Gaion F, Oliverio G, Duro M (2016). A randomized trial of intensive versus minimal surveillance of patients with resected dukes B2-C colorectal carcinoma. Ann Oncol.

[2] Wille-Jorgensen P, Syk I, Smedh K, Laurberg S, Nielsen DT, Petersen SH, Renehan AG, Horvath-Puho E, Pahlman L, Sorensen HT (2018). Effect of more vs less frequent follow-up testing on overall and colorectal Cancer-specific mortality in patients with stage II or III colorectal Cancer: the COLOFOL randomized clinical trial. JAMA.

[3] Pugh SA, Shinkins B, Fuller A, Mellor J, Mant D, Primrose JN (2016). Site and stage of colorectal Cancer influence the likelihood and distribution of disease recurrence and Postrecurrence survival: data from the FACS randomized controlled trial. Ann Surg.

[4] Snyder RA, Hu CY, Cuddy A, Francescatti AB, Schumacher JR, Van Loon K, You YN, Kozower BD, Greenberg CC, Schrag D (2018). Association between intensity of Posttreatment surveillance testing and detection of recurrence in patients with colorectal Cancer. JAMA.

[5] Meyerhardt JA, Mangu PB, Flynn PJ, Korde L, Loprinzi CL, Minsky BD, Petrelli NJ, Ryan K, Schrag DH, Wong SL (2013). Follow-up care, surveillance protocol, and secondary prevention measures for survivors of colorectal cancer: American Society of Clinical Oncology clinical practice guideline endorsement. J Clin Oncol.

[6] National Comprehensive Cancer Network: NCCN Clinical Practice Guidelines in Oncology: Colon Cancer. Obtained from: https://www.nccn.org/professionals/physician_gls/default.aspx. Accessed 8 Nov 2017. 10.6004/jnccn.2009.0056 19755046

[7] Steele SR, Chang GJ, Hendren S, Weiser M, Irani J, Buie WD, Rafferty JF (2015). Clinical practice guidelines Committee of the American Society of C, Rectal S: practice guideline for the surveillance of patients after curative treatment of Colon and Rectal Cancer. Dis Colon Rectum.

[8] Kievit J (2002). Follow-up of patients with colorectal cancer: numbers needed to test and treat. Eur J Cancer.

[9] Ohlsson B, Breland U, Ekberg H, Graffner H, Tranberg KG (1995). Follow-up after curative surgery for colorectal carcinoma. Randomized comparison with no follow-up. Dis Colon Rectum.

[10] Pietra N, Sarli L, Costi R, Ouchemi C, Grattarola M, Peracchia A (1998). Role of follow-up in management of local recurrences of colorectal cancer: a prospective, randomized study. Dis Colon Rectum.

[11] Secco GB, Fardelli R, Gianquinto D, Bonfante P, Baldi E, Ravera G, Derchi L, Ferraris R (2002). Efficacy and cost of risk-adapted follow-up in patients after colorectal cancer surgery: a prospective, randomized and controlled trial. Eur J Surg Oncol.

[12] Jeffery M, Hickey BE, Hider PN. Follow-up strategies for patients treated for non-metastatic colorectal cancer. Cochrane Database Syst Rev. 2007;1:CD002200. 10.1002/14651858.CD002200.pub2 17253476

[13] Tjandra JJ, Chan MK (2007). Follow-up after curative resection of colorectal cancer: a meta-analysis. Dis Colon Rectum.

[14] Kjeldsen BJ, Kronborg O, Fenger C, Jorgensen OD (1997). The pattern of recurrent colorectal cancer in a prospective randomised study and the characteristics of diagnostic tests. Int J Color Dis.

[15] Rose J, Augestad KM, Cooper GS (2014). Colorectal cancer surveillance: what's new and what's next. World J Gastroenterol.

[16] van der Stok EP, Spaander MCW, Grunhagen DJ, Verhoef C, Kuipers EJ (2017). Surveillance after curative treatment for colorectal cancer. Nat Rev Clin Oncol.

[17] Hines RB, Jiban MJH, Choudhury K, Loerzel V, Specogna AV, Troy SP, Zhang S (2018). Post-treatment surveillance testing of patients with colorectal cancer and the association with survival: protocol for a retrospective cohort study of the surveillance, epidemiology, and end results (SEER)-Medicare database. BMJ Open.

[18] Baca B, Beart RW, Etzioni DA (2011). Surveillance after colorectal cancer resection: a systematic review. Dis Colon Rectum.

[19] Koo SL, Wen JH, Hillmer A, Cheah PY, Tan P, Tan IB (2013). Current and emerging surveillance strategies to expand the window of opportunity for curative treatment after surgery in colorectal cancer. Expert Rev Anticancer Ther.

[20] Pal S (2014). Surveillance after colon cancer surgery: too much of a good thing?. In: *The ASCO Post*.

[21] Jeffery M, Hickey BE, Hider PN, See AM. Follow-up strategies for patients treated for non-metastatic colorectal cancer. Cochrane Db Syst Rev. 2016;(11). Art. No.: CD002200. 10.1002/14651858.CD002200.pub3. 10.1002/14651858.CD002200.pub3 PMC6464536 27884041

[22] Papagrigoriadis S (2007). Follow-up of patients with colorectal cancer: the evidence is in favour but we are still in need of a protocol. Int J Surg.

[23] Hershman DL, Wright JD (2012). Comparative effectiveness research in oncology methodology: observational data. J Clin Oncol.

[24] Lyman GH, Levine M (2012). Comparative effectiveness research in oncology: an overview. J Clin Oncol.

[25] Gionfriddo MR, Leppin AL, Brito JP, Leblanc A, Shah ND, Montori VM (2013). Shared decision-making and comparative effectiveness research for patients with chronic conditions: an urgent synergy for better health. J Comp Eff Res.

[26] Institue of Medicine (2009). Initial National Priorities for Comparative Effectiveness Research.

[27] Charlson ME, Pompei P, Ales KL, MacKenzie CR (1987). A new method of classifying prognostic comorbidity in longitudinal studies: development and validation. J Chronic Dis.

[28] Klabunde CN, Legler JM, Warren JL, Baldwin LM, Schrag D (2007). A refined comorbidity measurement algorithm for claims-based studies of breast, prostate, colorectal, and lung cancer patients. Ann Epidemiol.

[29] Sargent D, Sobrero A, Grothey A, O'Connell MJ, Buyse M, Andre T, Zheng Y, Green E, Labianca R, O'Callaghan C (2009). Evidence for cure by adjuvant therapy in colon cancer: observations based on individual patient data from 20,898 patients on 18 randomized trials. J Clin Oncol.

[30] National Comprehensive Cancer Network (2017). Clinical practice guidelines in oncology (NCCN guidelines®): Colon Cancer. Version 2.2017.

[31] Armstrong K (2012). Methods in comparative effectiveness research. J Clin Oncol.

[32] McCaffrey DF, Griffin BA, Almirall D, Slaughter ME, Ramchand R, Burgette LF (2013). A tutorial on propensity score estimation for multiple treatments using generalized boosted models. Stat Med.

[33] Friedman JH (2001). Greedy function approximation: a gradient boosting machine. Ann Stat.

[34] Friedman JH (2002). Stochastic gradient boosting. Comput Stat Data An.

[35] McCaffrey DF, Ridgeway G, Morral AR (2004). Propensity score estimation with boosted regression for evaluating causal effects in observational studies. Psychol Methods.

[36] U.S. Department of Commerce. Economics and Statistics Administration. U.S. Census Bureau. Census Regions and Divisions of the United States. Obtained from: https://www.census.gov/prod/1/gen/95statab/preface.pdf. Accessed 12 June 2018.

[37] Bohm B, Schwenk W, Hucke HP, Stock W (1993). Does methodic long-term follow-up affect survival after curative resection of colorectal carcinoma?. Dis Colon Rectum.

[38] Safi F, Link KH, Beger HG (1993). Is follow-up of colorectal cancer patients worthwhile?. Dis Colon Rectum.

[39] Kronborg O (1986). Controversies in follow-up after colorectal carcinoma. Theor Surg.

[40] Figueredo A, Rumble RB, Maroun J, Earle CC, Cummings B, McLeod R, Zuraw L, Zwaal C (2003). Gastrointestinal Cancer disease site Group of Cancer Care Ontario's program in evidence-based C: follow-up of patients with curatively resected colorectal cancer: a practice guideline. BMC Cancer.

[41] Jeffery GM, Hickey BE, Hider P. Follow-up strategies for patients treated for non-metastatic colorectal cancer. Cochrane Database Syst Rev. 2002;(1). Art. No.: CD002200. 10.1002/14651858.CD002200. 10.1002/14651858.CD002200 11869629

[42] Mokhles S, Macbeth F, Farewell V, Fiorentino F, Williams NR, Younes RN, Takkenberg JJM, Treasure T (2016). Meta-analysis of colorectal cancer follow-up after potentially curative resection. Brit J Surg.

[43] Renehan AG, Egger M, Saunders MP, O'Dwyer ST (2002). Impact on survival of intensive follow up after curative resection for colorectal cancer: systematic review and meta-analysis of randomised trials. BMJ.

[44] Primrose JN, Perera R, Gray A, Rose P, Fuller A, Corkhill A, George S, Mant D, Investigators FT (2014). Effect of 3 to 5 years of scheduled CEA and CT follow-up to detect recurrence of colorectal cancer: the FACS randomized clinical trial. JAMA.

[45] Jorgensen ML, Young JM, Solomon MJ (2015). Optimal delivery of colorectal cancer follow-up care: improving patient outcomes. Patient Relat Outcome Meas.

[46] Buie WD, Attard JA (2005). Follow-up recommendations for colon cancer. Clin Colon Rectal Surg.

[47] Sitlinger AP, Zafar SY (2018). With colorectal Cancer treatment, physical toxicity is not the only concern. Dis Colon Rectum.

[48] Yousuf Zafar S. (2015). Financial Toxicity of Cancer Care: It’s Time to Intervene. Journal of the National Cancer Institute.

[49] Berian JR, Cuddy A, Francescatti AB, O'Dwyer L, Nancy You Y, Volk RJ, Chang GJ (2017). A systematic review of patient perspectives on surveillance after colorectal cancer treatment. J Cancer Surviv.

