# Supplementary material for: The association between post-treatment surveillance testing and survival in stage II and III colon cancer patients: An observational comparative effectiveness study
Source: BMC Cancer. 2019 May 3;19:418. doi: 10.1186/s12885-019-5613-5 (PMC6500008; doi:10.1186/s12885-019-5613-5)
Supplement: Supplementary file 1 — Table S1. ICD-O-3, ICD-9/10-CM, CPT, HCPCS, and Revenue Center Codes. (DOCX 13 kb) [file 12885_2019_5613_MOESM1_ESM.docx]

| **Table S1** ICD-O-3, ICD-9/10-CM, CPT, HCPCS, and Revenue Center Codes | |
| --- | --- |
| Code type | Codes |
| ICD-O-3 Site codes | C18.0, C18.2-C18.9 |
| ICD-9 Colon cancer diagnosis codes | 153, 153.0-153.4, 153.6-153.9 |
| Surgery codes |  |
| ICD-9-CM | 17.31-17.36, 17.39, 45.30-45.34, 45.41-45.43, 45.49, 45.50-45.52, 45.61-45.63, 45.70-45.76, 45.79, 45.80-45.83, 45.90-45.95 |
| CPT codes | 44140-44141, 44143-44147, 44150-44158, 44160, 44204-44208, 44210-44212 |
| Chemotherapy codes |  |
| ICD-9-CM diagnosis codes | V58.1x, V66.2, V67.2 |
| ICD-9-CM procedure codes | 99.25 |
| CPT codes | 964xx, 96401-96402, 96409, 96411, 96413, 96415-96417, 965xx |
| HCPCS codes | C9205, C9214, C9257, G0345-G0351, G0353-G0363, J0610, J0640, J0641, J7150, J8499, J8510, J8520, J8521, J8530-J8999, J9000-J9999, Q0083-Q0085, Q2024, S0116 |
| Revenue Center codes | 0331, 0332, 0335 |
| National Drug Codes | 00004-1100-13, 00004-1100-20, 00004-1100-22, 00004-1100-23, 00004-1100-51, 00004-1101-13, 00004-1101-16, 00004-1101-50, 00004-1101-51, 00093-7473, 00093-7473-06, 00093-7474, 00093-7474-89, 00179-0149, 00179-0149-70, 00179-0195, 00179-0195-70, 00378-2511, 00378-2511-91, 00378-2512, 00378-2512-78, 16714-0467, 16714-0467-01, 16714-0468, 6714-0468-01, 16729-0072, 16729-0073, 16729-0073-29, 16729-072-12, 42291-0190, 42291-0191, 42291-019-12, 42291-019-60, 51079-0510, 51079-051-05, 60687-0149, 60687-014-94 |
| Surveillance codes |  |
| CEA |  |
| CPT codes | 82378 |
| Colonoscopy |  |
| ICD-9-CM | 45.23, 45.25 |
| CPT codes | 44388-44394, 44397, 45355, 45378, 45379, 45380-45393, 45398 |
| HCPCS codes | G0105, G0121 |
| Computed tomography codes |  |
| ICD-9-CM | 87.41, 88.01, 88.38 |
| CPT codes | 71250, 71260, 71270, 71275, 72191-72194, 74150, 74160, 74170, 74175-74178 |
| Positron emission tomography codes |  |
| CPT codes | 78811-78816 |
| HCPCS | G0213-G0215, G0163, G0231 |
| ICD-10 Cancer-related death codes | C00-C97 |
| Abbreviations: ICD-O-3, International Classification of Diseases for Oncology, 3rd Edition; ICD-9/10-CM, International Classification of Diseases, 9^th^/10^th^ Edition, Clinical Modification; CPT, Current Procedural Terminology; HCPCS, Healthcare Common Procedure Coding System. | |
