# Supplementary material for: The association between post-treatment surveillance testing and survival in stage II and III colon cancer patients: An observational comparative effectiveness study
Source: BMC Cancer. 2019 May 3;19:418. doi: 10.1186/s12885-019-5613-5 (PMC6500008; doi:10.1186/s12885-019-5613-5)
Supplement: Supplementary file 2 — Table S2. Classification scheme for overall surveillance assessments. (DOCX 12 kb) [file 12885_2019_5613_MOESM2_ESM.docx]

| **Table S2** Classification scheme for overall surveillance assessments | | |
| --- | --- | --- |
| Surveillance for Patients followed 2 - < 3 years | | |
| Years 1 and 2 Categorization | Year 1 Categorization | Year 2 Categorization |
| More Adherent | More Adherent | More Adherent |
|  | More Adherent | Less Adherent |
|  | Less Adherent | More Adherent |
| Less Adherent | More Adherent | Nonadherent |
|  | Nonadherent | More Adherent |
|  | Less Adherent | Less Adherent |
|  | Less Adherent | Nonadherent |
|  | Nonadherent | Less Adherent |
| Nonadherent | Nonadherent | Nonadherent |
| Surveillance for Patients followed ≥ 3 years | | |
| Years 1, 2, and 3 Categorization | Years 1 & 2 Categorization | Year 3 Categorization |
| More Adherent | More Adherent | More Adherent |
|  | More Adherent | Less Adherent |
|  | Less Adherent | More Adherent |
| Less Adherent | More Adherent | Nonadherent |
|  | Less Adherent | Less Adherent |
|  | Nonadherent | More Adherent |
|  | Nonadherent | Less Adherent |
|  | Less Adherent | Nonadherent |
| Nonadherent | Nonadherent | Nonadherent |
