# Supplementary material for: The association between post-treatment surveillance testing and survival in stage II and III colon cancer patients: An observational comparative effectiveness study
Source: BMC Cancer. 2019 May 3;19:418. doi: 10.1186/s12885-019-5613-5 (PMC6500008; doi:10.1186/s12885-019-5613-5)
Supplement: Supplementary file 3 — Table S3. Unweighted and Weighted Means According to Adherence Status and the Unweighted Study Population. (DOCX 19 kb) [file 12885_2019_5613_MOESM3_ESM.docx]

| **Table S3** Unweighted and Weighted Means According to Adherence Status and the Unweighted Study Population | | | | | | |
| --- | --- | --- | --- | --- | --- | --- |
|  | Unweighted means | | Weighted means | | Study Population | |
| Characteristic | More Adherent | Less Adherent | More Adherent | Less Adherent | Mean | SD |
| Unweighted, weighted sample size (n, %) | 11840 (66.3) | 6020 (33.7) | 17785.5 (50.4) | 17471.1 (49.6) | 17860 |  |
| Age at diagnosis |  |  |  |  |  |  |
| 66-74 years | 0.491 | 0.351 | 0.444 | 0.437 | 0.444 | 0.497 |
| 75-79 years | 0.289 | 0.295 | 0.290 | 0.291 | 0.291 | 0.454 |
| 80-84 years | 0.220 | 0.354* | 0.266 | 0.271 | 0.265 | 0.442 |
| Race |  |  |  |  |  |  |
| White | 0.849 | 0.842 | 0.846 | 0.845 | 0.846 | 0.361 |
| Black | 0.080 | 0.094 | 0.085 | 0.086 | 0.084 | 0.278 |
| Asian | 0.036 | 0.027 | 0.033 | 0.031 | 0.033 | 0.178 |
| Other | 0.020 | 0.016 | 0.019 | 0.019 | 0.019 | 0.136 |
| Hispanic | 0.013 | 0.017 | 0.015 | 0.016 | 0.014 | 0.119 |
| Native American | 0.002 | 0.004 | 0.002 | 0.003 | 0.003 | 0.051 |
| Unknown | 0.001 | 0.001 | 0.001 | 0.001 | 0.001 | 0.026 |
| Sex |  |  |  |  |  |  |
| Male | 0.439 | 0.447 | 0.440 | 0.444 | 0.441 | 0.497 |
| Marital status |  |  |  |  |  |  |
| Married or partner | 0.581 | 0.484 | 0.549 | 0.543 | 0.548 | 0.498 |
| Separated/divorced | 0.065 | 0.073 | 0.067 | 0.066 | 0.067 | 0.250 |
| Single | 0.072 | 0.089 | 0.078 | 0.078 | 0.078 | 0.268 |
| Widowed | 0.254 | 0.315 | 0.276 | 0.277 | 0.275 | 0.446 |
| Unknown | 0.000 | 0.000 | 0.000 | 0.000 | 0.000 | 0.000 |
| Year of diagnosis |  |  |  |  |  |  |
| 2002-2003 | 0.288 | 0.302 | 0.293 | 0.294 | 0.293 | 0.455 |
| 2004-2006 | 0.375 | 0.383 | 0.379 | 0.383 | 0.378 | 0.485 |
| 2007-2009 | 0.337 | 0.315 | 0.328 | 0.323 | 0.329 | 0.470 |
| State buy-in coverage |  |  |  |  |  |  |
| Yes | 0.191 | 0.285 | 0.223 | 0.228 | 0.223 | 0.416 |
| Census tract poverty level |  |  |  |  |  |  |
| Low | 0.279 | 0.248 | 0.268 | 0.263 | 0.268 | 0.443 |
| Lower-middle | 0.278 | 0.271 | 0.275 | 0.271 | 0.275 | 0.447 |
| Upper-middle | 0.273 | 0.287 | 0.278 | 0.283 | 0.277 | 0.448 |
| High | 0.168 | 0.191 | 0.176 | 0.179 | 0.176 | 0.381 |
| Unknown | 0.003 | 0.003 | 0.003 | 0.003 | 0.003 | 0.056 |
| Geographic residency |  |  |  |  |  |  |
| Urban | 0.871 | 0.874 | 0.872 | 0.872 | 0.872 | 0.334 |
| Less Urban | 0.103 | 0.104 | 0.103 | 0.104 | 0.103 | 0.305 |
| Rural | 0.025 | 0.021 | 0.024 | 0.024 | 0.024 | 0.153 |
| Unknown | 0.000 | 0.000 | 0.000 | 0.000 | 0.000 | 0.013 |
| SEER Region |  |  |  |  |  |  |
| West | 0.357 | 0.368 | 0.361 | 0.362 | 0.361 | 0.480 |
| South | 0.261 | 0.258 | 0.261 | 0.266 | 0.260 | 0.439 |
| Northeast | 0.220 | 0.216 | 0.218 | 0.213 | 0.218 | 0.413 |
| Midwest | 0.148 | 0.146 | 0.147 | 0.146 | 0.147 | 0.354 |
| Pacific | 0.015 | 0.012 | 0.013 | 0.013 | 0.014 | 0.116 |
| Disease Stage |  |  |  |  |  |  |
| Stage II | 0.499 | 0.680* | 0.559 | 0.572 | 0.560 | 0.496 |
| Stage III | 0.501 | 0.320* | 0.441 | 0.428 | 0.440 | 0.496 |
| Tumor grade |  |  |  |  |  |  |
| Low grade | 0.744 | 0.799 | 0.762 | 0.766 | 0.763 | 0.425 |
| High grade | 0.235 | 0.184 | 0.218 | 0.216 | 0.218 | 0.413 |
| Unknown | 0.021 | 0.017 | 0.020 | 0.019 | 0.019 | 0.138 |
| Tumor site |  |  |  |  |  |  |
| Proximal colon | 0.644 | 0.655 | 0.648 | 0.646 | 0.648 | 0.478 |
| Distal colon | 0.356 | 0.345 | 0.352 | 0.354 | 0.352 | 0.478 |
| Adjuvant chemotherapy | 0.534* | 0.195* | 0.421 | 0.408 | 0.420 | 0.494 |
| Myocardial infarction | 0.051 | 0.068 | 0.056 | 0.056 | 0.057 | 0.253 |
| Congestive heart failure | 0.102 | 0.164 | 0.122 | 0.127 | 0.123 | 0.328 |
| Peripheral vascular disease | 0.076 | 0.097 | 0.083 | 0.084 | 0.083 | 0.276 |
| Cardiovascular disease | 0.052 | 0.083 | 0.062 | 0.064 | 0.063 | 0.242 |
| Chronic obstructive pulmonary disease | 0.162 | 0.195 | 0.173 | 0.180 | 0.173 | 0.378 |
| Dementia | 0.013 | 0.038 | 0.021 | 0.022 | 0.021 | 0.144 |
| Paralysis | 0.003 | 0.010 | 0.005 | 0.006 | 0.006 | 0.075 |
| Diabetes without complications | 0.238 | 0.258 | 0.244 | 0.247 | 0.244 | 0.430 |
| Diabetes with complications | 0.056 | 0.075 | 0.062 | 0.062 | 0.062 | 0.241 |
| Renal disease | 0.038 | 0.054 | 0.043 | 0.043 | 0.043 | 0.204 |
| Mild liver disease | 0.009 | 0.011 | 0.009 | 0.010 | 0.009 | 0.096 |
| Moderate/severe liver disease | 0.002 | 0.002 | 0.002 | 0.002 | 0.002 | 0.048 |
| Ulcers | 0.023 | 0.029 | 0.024 | 0.024 | 0.025 | 0.156 |
| Rheumatic disease | 0.021 | 0.019 | 0.020 | 0.019 | 0.021 | 0.142 |
| AIDS | 0.000 | 0.000 | 0.000 | 0.000 | 0.000 | 0.015 |
| *Indicates an absolute standardized mean difference of ≥ 0.20. | | | | | | |
